# The Threshold Bias Model: A Mathematical Model for the Nomothetic Approach of Suicide

**DOI:** 10.1371/journal.pone.0024414

**Published:** 2011-09-01

**Authors:** Walter Sydney Dutra Folly

**Affiliations:** NIPPEC - Núcleo Integrado de Pesquisa e Pós-Graduação em Educação e Ciências Universidade Federal de Sergipe, SE, Brazil; Durham University, United Kingdom

## Abstract

**Background:**

Comparative and predictive analyses of suicide data from different countries are difficult to perform due to varying approaches and the lack of comparative parameters.

**Methodology/Principal Findings:**

A simple model (the Threshold Bias Model) was tested for comparative and predictive analyses of suicide rates by age. The model comprises of a six parameter distribution that was applied to the USA suicide rates by age for the years 2001 and 2002. Posteriorly, linear extrapolations are performed of the parameter values previously obtained for these years in order to estimate the values corresponding to the year 2003. The calculated distributions agreed reasonably well with the aggregate data. The model was also used to determine the age above which suicide rates become statistically observable in USA, Brazil and Sri Lanka.

**Conclusions/Significance:**

The Threshold Bias Model has considerable potential applications in demographic studies of suicide. Moreover, since the model can be used to predict the evolution of suicide rates based on information extracted from past data, it will be of great interest to suicidologists and other researchers in the field of mental health.

## Introduction

The first mathematical model of age-specific mortality in humans was proposed by Benjamin Gompertz in 1825 [1]. He attributed the mortality rates to age-specific factors that follow a double exponential function of age. This model is still in use today, albeit it does not take into account external factors and other potentially important contributions including suicide. Indeed, the lack of specific mathematical models to deal with suicide data is one of the many problems in the field of Suicidology.

Suicidologists often face difficulties when performing comparative analyses of suicide rates from different countries. Such difficulties may be caused by undercounting [2], rapid demographic changes [3], misclassification [4] and by problems attributable to data reporting. For example, the use of wide age groups (e.g. comprising ten or more years) to report death rates by age can cause information loss due to data inhomogeneity within these age groups. To avoid this, some authors occasionally have reported death rates by age in a more detailed way [5], [6]. In response to some of these issues we recently proposed a simple mathematical model to deal with suicide rates by age [7]. This model (called the Threshold Bias Model), allows the fitting of age-specific suicide rates by a piecewise distribution that can be used in rate predictions, comparative studies of suicide data and various types of demographic studies, including procedures to estimate the completeness of death registration as reported by other authors [8].

The objective of the present study is to provide a detailed suicidology-based explanation of the Threshold Bias Model (TBM), and to illustrate its application through a comparative analysis of suicide data sets from three different countries.

### The Threshold Bias Model

The field of Suicidology was strongly influenced by theoretical conceptions attributed to Emile Durkheim and Sigmund Freud. While Durkheim regarded suicidal behaviors in terms of moral regulation and social integration forces (external causes), Freud considered them a consequence of the action of unconscious death drives (internal causes) against the subject himself.

Starting from Durkheim's assertion that “…the term suicide is applied to all cases of death resulting directly or indirectly from a positive or negative act of the victim himself, which he knows will produce this result” [9], we are led to consider suicide as a conscious act, which requires certain level of discernment. In line with this, de Catanzaro [10] remarked that suicide requires a threshold level of intelligence, which allows the individual to be self-aware of his existence in a context of suffering.

From the perspective of Psychoanalytic Theory, aggressive behaviors (including suicide and suicide attempts) are related to ‘death drives’ acting in a partially isolated form [11]. Normally, ‘death drives’ are counterbalanced by ‘life drives’ in different proportions and are never found in a totally isolated form [12].

In his initial theory, Freud conceived consciousness (named system 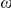) as being the surface of the psyche [13]. Therefore, it receives stimuli from the external reality via sensory organs (quantity 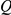) and from the interior of the organism (quantity 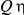). Beyond this, Freud considered that part of quantity 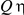 can be associated to the pressure (*drang*) of the drives, which act as a propelling agent for the psyche [14]. Under this framework the pressures caused by drives are always present and cannot be avoided by the individual, contrary to his/her perception of external stimuli, which can be consciously controlled.

Several contemporary authors have reported increased impulsivity as a precursor of suicidal behaviors [15], [16], including suicide attempts. According to O'Carroll et al. [17], a suicide attempt is defined as a self-destructive behavior with the intent to end one's life, independent of resulting damage. In a recent study, Baca-Garcia et al. [18] used Beck's Suicidal Intent Scale (SIS) to investigate suicide attempts with respect to the dimensions of lethality, planning and attempt impulsivity. They observed that planning appears to be the opposite dimension of impulsivity. In this same study, they found an inverse relation between attempt impulsivity and lethality, a result that corroborates the findings of previous studies [19]. Although such investigations are based on analyses of suicide attempts, it is well known in the field of Suicidology that there is a direct relation between suicide attempts and completed suicides [20], [21]. Thus, considering these findings, we may suppose that suicide rates by age follow a distribution 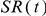 defined by the product between a self-destructive impulsivity factor (inversely related to lethality) and a consciousness factor (directly related to planning):
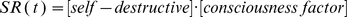



Moreover, we can consider the self-destructing impulsivity factor as linked to the Freudian concept of ‘death drive’. Since suicides are triggered by threshold factors [22], [23], we can envisage 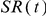 as a piecewise distribution whose non-zero piece is the product of two functions of age 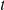: The threshold bias function 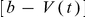, which represents the self-destructive impulsivity, and the consciousness function 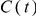. Thus, 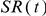 can be defined as:
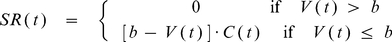
(1)Where 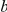 is the threshold level and 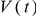 is a representation of the fusion of ‘life drives’ and ‘death drives’. In reality, we assume that these two categories of drives may contribute at different proportions during the individual's lifetime oscillating around its average value given by 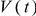. Therefore, a suicide attempt only occurs when the fused drives assume a certain value, which is shifted from its average value 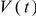 by the constant 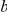. In other words, the pressure (*drang*) of ‘death drives’ needs to reach this threshold in order to trigger the suicidal behavior. Once the behavior is triggered, Equation 1 provides the probability that the self-destructing act will result in death as increasing with the factor 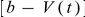 modulated by the factor 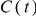.

Considering the consciousness level and the fused drives as represented by 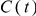 and 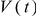, the Freudian concepts previously discussed enable us to assume the rate of change of the consciousness level as given by the product of these two functions. This conjecture can be explained as follows: The more aware an individual is, the more vivid is his/her perception of external stimuli and the faster his/her consciousness level evolves with time under the action of the drives. Mathematically, this can be expressed as:
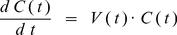
(2)Solving the Equation 2 for 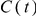 and replacing the result in the Equation 1, we have:
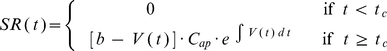
(3)Where 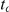 is the critical age obtained by solving the equation 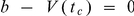 and 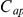 is the consciousness apex constant, which arises from the integration of Equation 2. While 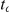 can be easily interpreted as the age below which suicides do not occur (or are considered statistically rare), 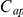 is a constant that represents the average apex of mental skills (potentially associated with suicide planning) observed in the studied cohort.

Due to the great complexity of the psychoanalytic concept of drive, it can only be taken as a conceptual inspiration and mathematically modeled in a very simplified way. In his letter addressed to Einstein [24], Freud pointed out an aspect of the drive dualism that is sufficiently simple to be used in mathematical modeling: The idea of the antagonism between ‘life drives’ and ‘death drives’ as being compared with attractive or repulsive forces in Physics.

Considering the life and death drives always act together, as well as the dualistic concept of drive, we can define the average drive function 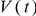 as being a decreasing function of age, which assumes positive values during youth, near to zero values at intermediary ages (with an inflection point at the middle age) and negative values during old ages [7]. Upon these considerations, we can define 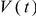 by the following simple function:
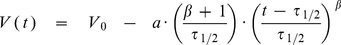
(4)where 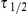 is the middle age; 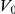 is a quiescent drive constant (which defines the value assumed by the average drive function when 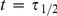); 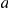 is a maturational constant and the exponent 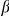 (which is also related to maturational processes) can be any odd integer starting from 1. Thus, from a mathematical perspective, 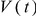 can assume the simplest form of a straight line with negative slope (when 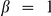). Nevertheless, since neurological and psychological maturation are not linearly correlated with age, we should expect values of 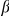 considerably higher than 1.

Replacing 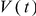 as given by the Equation 4 in Equation 3, 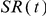 can be rewritten as follows [7]:
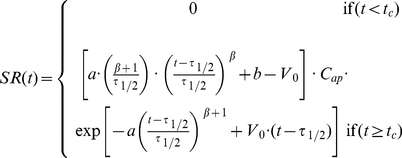
(5)Considering that 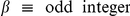, the critical age is given by the following expression:
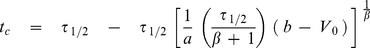
(6)


At this point, we should note that the main properties of Equation 5 are defined by the chosen average drive function 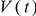. In reality, we could choose any other form of 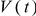 with same previously discussed properties. In the present case, the form expressed in Equation 4 was motivated by simplicity. Using this choice, Equation 5 tends to the well-known Weibull distribution [25] with shape parameter 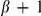 when both the parameters 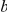 and 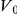 tend to zero. Moreover, the so-called threshold parameter 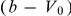 may be adopted as a direct indicator of average suicidal impulsivity. Thus, at middle age (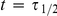), the suicide rate is obtained by evaluating the product between 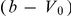 and the apex constant 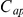.

In order to demonstrate the utility of the TBM in nomothetic studies of suicide, it was applied in two different situations: The predictive analyses of annual suicide rates from a specific country (USA) for the years 2001 [5], 2002 [6] and 2003 [26], and the comparative study of suicide rates in early adolescence in the USA, Brazil (2002) [27] and Sri Lanka (1999) [28].

## Results

**Figure 1:**
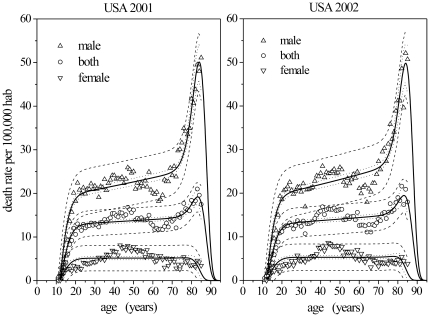
Suicide rates by age and gender in USA referring to the years 2001 and 2002. The data points marked with circles represent the average rate (considering both genders). The curves shown in continuous lines were obtained by fitting the Equation 5 over the aggregate data. Dashed and dotted curves define, respectively, the prediction bands and the confidence bands calculated for 95% confidence intervals.

All data analyses were performed using the software Microcal Origin version 5.0 [29] (although the newest versions of this software or other similar programs may also be used). In a first step, we fitted the function 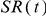 to the USA data sets for the years 2001 and 2002. Rapid visual inspections of these graphs revealed that male suicide rates by age shows a characteristic “N” shaped curve, which is associated with higher suicide rates at older ages (Figure 1). In order to facilitate comparisons, the parameter values found in these fitting sections were displayed in a table with the corresponding degrees of freedom, chi-square values (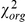 and 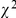), as well as the two-tailed p-values (Table 1). The dashed and dotted curves shown in the Figure 1 define, respectively, the prediction bands and the confidence bands for a 95% confidence interval. For the female data sets it was necessary to keep fixed values of 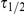 and 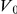 in order to allow the convergence of fitting process.

**Table 1:** TBM's parameter values with respective standard errors.

Country	Year	Gender	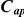	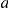	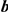 (years^ –1^)	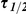 (years)	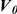 (years^ –1^)	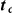 (years)	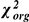	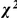	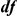	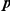
Brazil	2002 *	Males	60.2 (7.1)	17.8 (2.4)	0.189 (0.023)	46	0.0053 (0.0015)	11.7 (0.6)	0.94	1.076	11	>0.999
		Females	15.9 (6.4)	11.3 (4.8)	0.165 (0.070)	47	-0.001 (0.002)	10.7 (2.0)	0.12	0.495	11	>0.999
Sri Lanka	1999 *	Males	737 (108)	13.9 (3.3)	0.093 (0.016)	42.2 (0.6)	0	12.2 (0.8)	148.3	25.08	10	0.005
USA	2001 *	Males	287 (17)	8.20 (0.76)	0.0817 (0.0054)	46.30 (0.25)	0.00416 (0.00089)	12.0 (0.4)	6.76	21.37	68	>0.999
		Females	15.5 (3.7)	28.6 (6.1)	0.332 (0.082)	47	0	11.5 (1.1)	2.11	32.06	71	>0.999
		Both	94.3 (7.9)	15.2 (1.6)	0.147 (0.013)	47.49 (0.44)	0.00195 (0.00078)	12.2 (0.5)	2.10	10.42	68	>0.999
	2002 *	Males	271 (18)	9.19 (0.94)	0.0890 (0.0064)	46.56 (0.29)	0.00496 (0.00095)	12.2 (0.4)	8.17	23.60	68	>0.999
		Females	18.1 (4.3)	30.3 (6.9)	0.292 (0.070)	47	0	12.1 (1.1)	2.30	32.69	69	>0.999
		Both	91.4 (8.9)	16.8 (2.0)	0.155 (0.016)	47.69 (0.53)	0.00247 (0.00088)	12.4 (0.6)	2.80	13.47	68	>0.999
	2003 **	Both	88.4 (9.6)	18.4 (2.4)	0.163 (0.019)	47.9 (0.6)	0.003 (0.001)	12.6 (0.6)	__	14.54	8	0.069

*Parameter values obtained by fitting.

**Parameter values obtained by linear extrapolation.

**Figure 2:**
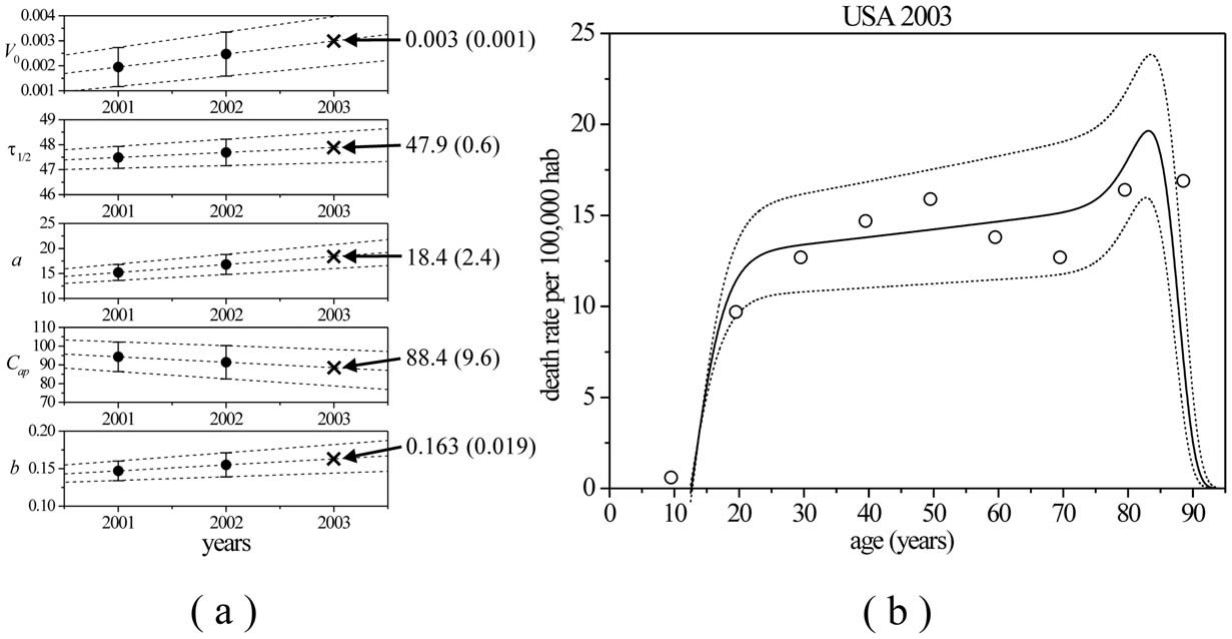
Suicide rates by age in USA averaged for both genders referring to the year 2003. (a) Linear extrapolations of parameter values obtained for the years 2001 and 2002 in order to estimate their values for 2003. (b) Prediction curve (continuous line) and the prediction bands (dashed lines) for the year 2003.

After obtaining the parameter values for 2001 and 2002 we performed linear extrapolations in order to determine their values for 2003 with the respective standard errors (Figure 2a). These values were then reintroduced into Equation 5 in order to calculate the expected curve for 2003 with prediction bands (Figure 2b) at one standard deviation (68.2% confidence interval). The TBM predictions are in reasonable agreement with the data and the differences are not significant (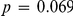).

**Figure 3:**
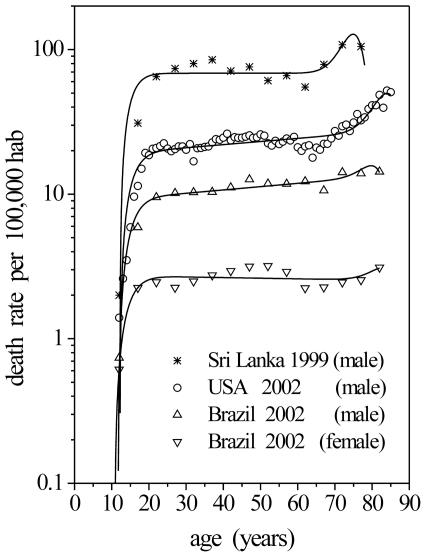
Semi-log graph of suicide rates by age. The curves shown as continuous lines were obtained by using the Equation 5 to fit the aggregate data from Sri Lanka (1999, males), USA (2002, males) and Brazil (2002, males and females). Note the tendency towards the same average critical age.

Suicide rates from USA (2002, males), Brazil (2002, males and females) and Sri Lanka (1999, males) were plotted together (Figure 3) using a logarithmic scale to improve the graphical presentation of the slope of these rates in early adolescence. Despite the enormous differences between the presented aggregate data, all curves tend to the same average value of critical age when standard deviations are considered. The values of TBM parameters obtained for these additional aggregate data with standard errors in parentheses are shown in Table 1. It should be noted that while the values of all parameters change with gender and year, the critical age remains close to the average value of 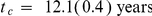. The differences between the TBM predictions and the analyzed data were significant only in the case of Sri Lanka (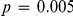) - a result attributable to the low number and high dispersion of data points.

**Figure 4:**
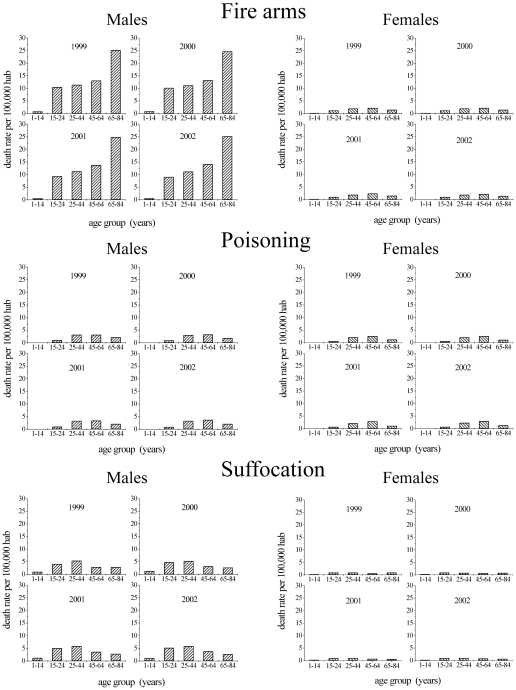
Suicide rates by age, gender and method in the USA in the quadrennium 1999-2002. This figure is a graphic representation of data sets reported in reference [6] (Table 17). While the male suicide rates are strongly related to firearms, female rates are not clearly associated with a specific suicide method.

A visual inspection of suicide rates between 1999 and 2002 (Figure 4) classified by method, gender and age (data extracted from [6], Table 17) reveals that firearms is the suicide method predominantly chosen by males. Females have no clear preference – showing only a slight tendency towards poisoning. We also observed that suicide rates associated to poisoning and suffocation reach their maxima at ages around 45 years for both genders. While suicide rates associated to these less lethal methods were characterized by similar trends, the rates associated to fire arms are enormously different for males and females. For males, suicide rates associated to fire arms increase strongly from 10 to 20 years old and in ages higher than 70. However, female suicide rates associated to fire arms show similar trends to less lethal methods.

## Discussion

The main assumption of the TBM is that suicides require a certain level of intelligence and self-consciousness. As explained in the introduction, this assumption is consistent with de Catanzaro's hypothesis [10], who stated that: “…it may take an intelligent animal to know when the situation is hopeless, to realize that purpose for life is removed in those circumstances, and that death can be self-induced”. This hypothesis was also recently explored by Martin Voracek, who observed a positive relation between national suicide rates and average IQ in several countries [30]. In addition, other authors have considered suicide as a conscious escape from painful self-awareness [31] and associations between mental strategies to suppress aversive thoughts and self-injury have been observed [32].

The average drive function 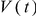 considered in the TBM was defined as being a decreasing function that passes through zero at certain age, assuming positive values below (‘life drives’ domain) and negative values above this age (‘death drives’ domain). This hypothesis may be plausible from a psychoanalytic viewpoint if we suppose that the ordering action of ‘life drives’ supplants the disordering action of ‘death drives’ from conception to early adulthood (when mental and sexual maturity is achieved). As considered by Freud (in his interview with Viereck [33]), the inverse situation of ‘death drives’ overcoming ‘life drives’ supposedly occurs at the end of individual’s lifetime. Freud is quoted as saying: “…Mankind does not choose suicide, because the law of its being abhors the direct route to its goal. Life must complete its cycle of existence. In every normal being, the life-wish is strong enough to counterbalance the death-wish, albeit in the end the death-wish proves stronger. We may entertain the fanciful suggestion that death comes to us by our own volition. It is possible that we could vanquish death, except for his ally in our bosom. In that sense, we may be justified in saying that all death is suicide in disguise…”. Thus, in Freud's view, the possibility of suicidal behavior is regarded as an innate characteristic.

The TBM predicts a peak in suicide rates in the oldest age groups since 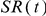 increases with the function 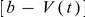 modulated by the exponential function 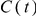. The increase in suicide rates until this peak can be explained as a consequence of increasing impulsivity and poor problem solving in the elderly [34], [35]. Nevertheless, as 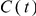 decays exponentially toward zero in the limit of very advanced ages, the same is expected to occur with predicted suicide rates 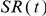 (Figures 1 and 2b). This occurs due to the interdependence between 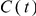 and 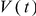 as described by the Equation 2, as well as the properties of 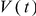. Therefore, this is a consequence of the choice of a power law of age 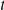 (with odd exponent) as stated in Equation 4. Due to the lack of statistical data for ages higher than 85, it is very difficult to verify how suicide rates decrease with age and whether they really tend to zero in the limit of very advanced ages. In Brazil, there have been very few studies that report this decrease (e. g. see [36]). However, it may be statistically expected as a consequence of progressive loss of cognitive skills associated with Alzheimer's disease and other neurological impairments often observed in the elderly. This finding emphasizes the importance of detailed demographic studies in order to reveal the trends of suicide rates at the upper limit of human lifespan.

Some authors have reported an inverse relation between lethality and attempt impulsivity, as well as between attempt impulsivity and planning [18], [19]. Consequently, we may expect a direct relation between lethality and planning. In our analysis of USA data sets, we found small values of threshold parameter 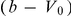 for males, intermediary values for both genders analyzed together, and large values for females (Table 1). Otherwise, for the apex constant 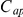, we found large values for males, intermediary values for both genders, and small values for females.

Considering that suicide rates for males are strongly related to high-lethality methods (firearms) (see Figure 4), the associations discussed above indicate that small values of 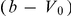 and large values of 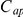 correspond to “N” shaped curves, high lethality methods, high planning and low impulsivity. However, it should be noted that female suicide rates attributed to firearms do not conform to this pattern (Figure 4) and that the model fits the data better for males than for females in all age ranges (Figures 1 and 3). These observations can be understood in the context of the finding that, in contrast to men, suicide methods chosen by women are very evenly distributed (Figure 4). Moreover, according with Stack and Wasserman [37], women are about 47% less likely to shoot themselves in the head than men. Such a fact leads us to consider that, on average, firearms have lower lethality in the way they are used by women. This explains the absence of the “N” shaped curves and the high values of 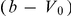 found in the analysis of female data. It should also be noted that the great majority of firearms in circulation in the USA are owned and used by men [38], which suggests that women with suicidal tendencies may face more difficulties gaining access to firearms than men. In countries where women have easy access to lethal methods, female suicide rates are very similar to male rates in all age groups [3].

Another remarkable result of the analysis is the coarse fit of the statistical data in the 35-65 age range, especially for ages around 45. As shown in Figure 4, there is a peak in suicide rates at this age due to the increased contribution of low lethality methods (poisoning and suffocation). Thus, if we consider that different methods have different lethalities and, consequently, different values of 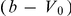 and 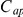, we may expect better fits when the model is applied to statistical data separated by methods. Unfortunately, detailed statistical data (such as the shown in Figure 1) are not available for separate methods thereby preventing the testing of the model under such conditions.

**Figure 5:**
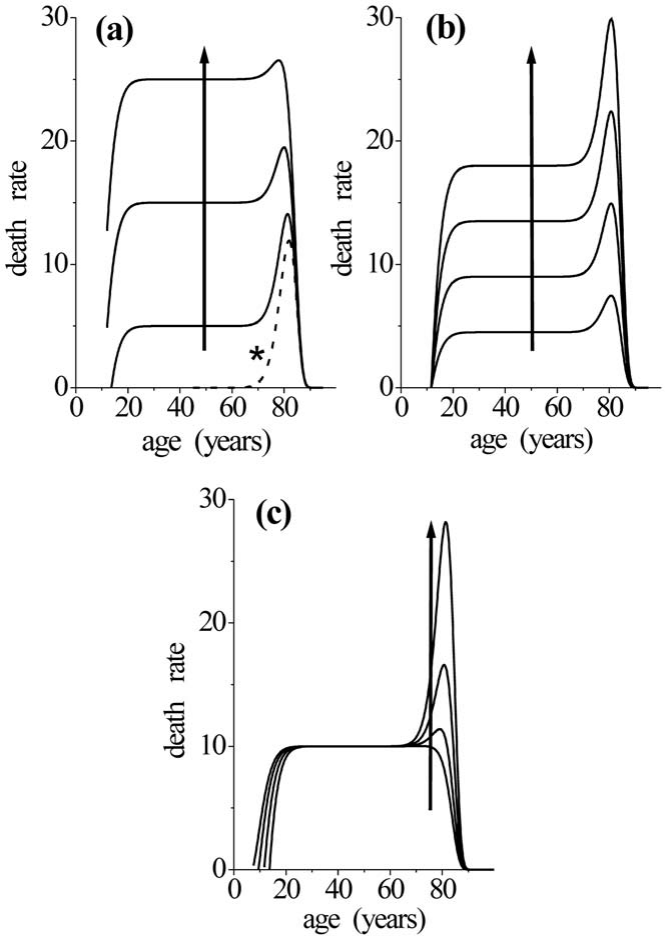
Influence of main TBM parameters on the obtained curve shapes. Distributions calculated for: (a) Increasing threshold parameter, apex constant fixed; (b) Increasing apex constant, threshold parameter fixed and (c) constant product between these two parameters. The curve marked with an asterisk in (a) is the Weibull distribution.

Distributions simulated for different values of threshold parameter and apex constant are shown in the Figure 5. If 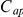 is kept constant, the variation of the threshold parameter 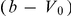 leads to remarkably different distributions (Figure 5a), which include the form proposed by Weibull [25] when this parameter is equal to zero (curve marked with an asterisk). However, as 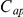 is a global multiplicative factor, we obtain similar curves when we vary this parameter keeping 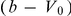 constant (Figure 5b). Figure 5c illustrates what happens when these two parameters are changed in way to keep the product between them constant.

In the context of Voracek's findings [30], it is plausible to suppose the existence of a positive relation between 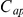 and the average IQ of the studied population. However, this hypothesis must be treated with caution. Although Voracek observed a positive relation between national IQ and average suicide rates, this has not been confirmed for a large number of countries. From the perspective of the TBM, a link between suicide and IQ could occur because suicidal behavior should be regarded as the result of dichotomous forces. Thus, suicide depends on conscious factors related to planning and intelligence and on unconscious factors related to impulsivity and drive.

Beyond the parameters 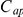 and 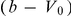, to which the model is more sensitive, we can tentatively infer the meaning of the other parameters as follows:

### Maturational constant (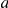) and exponent (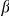)

These two parameters can be related to neurological and psychological maturation. An attempt to obtain the values of these parameters from neurological data was performed in a preliminary study [39]. Further studies have been conducted in order to verify this possibility.

### Middle age (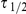)

This parameter is directly linked to the life expectancy of the studied population. Developed countries with good public health services and high life expectancy are normally characterized by larger values of 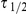. This fact may be related to increasing suicide rates of elderly people in these countries [40].

The average age at which suicide completion starts to be observed was estimated as 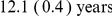. This result is consistent with the findings of other authors that have reported the onset of suicidal behaviors among adolescents at ages between 10 and 15 years [41]-[43]. According to Jamison [44], the onset of suicidal behaviors in this age range may be related to the average age of puberty, when depression often starts to occur. For ages immediately above 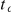, the consciousness function 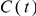 in the TBM is well below its apex. Thus, during early adolescence the model assumes that fewer suicides are related to planned acts. This hypothesis is corroborated by a recent study in South Africa, in which the authors suggested that unplanned suicide attempts are prevalent in the age range from 10 to 20 years [21]. Furthermore, these authors reported that the onset of suicide planning and ideation is typically associated with people in their late twenties.

Several authors have related suicidal behaviors with bipolar and rapid-cycling bipolar disorder [45], [46], and the hypotheses previously discussed are compatible with the sensitization/kindling model proposed by Post and colleagues [47], [48]. These authors consider that young individuals with untreated unipolar depression can develop rapid-cycling bipolar disorder as a consequence of repeating episodes of strong emotional stress across their lifetimes. Therefore, it is very important that parents, teachers and educators stay alert to any manifestation of suicidal ideation, depression and/or mood disorders at the stage of early adolescence. Suicidal ideation and self-destructive thoughts at these ages have also been associated with bullying [49]-[51].

In conclusion, the TBM is a potentially useful tool to predict annual suicide rates by extrapolation of parameter values found in preceding years. Moreover, its parameters can be used in comparative analyses of suicide rates from different countries. The TBM also provides a continuous mathematical distribution that can be used to estimate the completeness of suicide registration and other statistical calculations.

## Materials and Methods

The USA data sets used in the analyses relate to the leading causes of death reported for the years 2001 (reference [5], Table 11), 2002 (reference [6], Table 15) and 2003 (reference [26], Table 9). Similar data sets were used for Brazil (2002, male and female) and Sri Lanka (1999, male) to perform a comparative study of suicide rates in early adolescence. These data were obtained at the website of Brazilian Ministry of Health/DATASUS [27] and at the website of Department of Census and Statistics - Sri Lanka [28], respectively. All data sets referred in this research are in the public domain and no permission is necessary to reproduce them.

In order to allow the fitting of aggregate data, the function 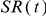 (given by the Equation 5) was edited in the “Non-linear curve fit” tool of Origin 5.0, which was used to fit curves to data points by the Levenberg-Marquardt method.

The program Origin requires an appropriate choice of the initial parameter values in order to start fitting sections. For this, we used the values shown in Material S1 [7], in which 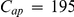; 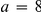; 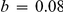; 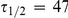. For 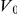, we arbitrarily choose the initial value 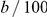. Other initial values may be tried considering that 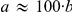, 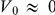 and that the product 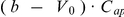 should fit the observed suicide rate at middle age 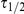. The initial value of middle age can be estimated as half of life expectancy observed for the studied population. As the value of the exponent 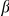 is necessarily an odd integer, it needs to be fixed by the program's user in way to minimize the standard errors of all parameters, the value of chi-square and the width of confidence bands. In the preliminary study cited above better results were achieved with 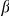 equal to 9 or 11 and, in the present work, we used 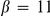. Researchers studying other population groups may obtain better results by using other values of 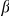 between 7 and 15. The convergence of the fitting occurs after a relatively small number of iterations, depending on the parameters initialization and the studied dataset. Considering the USA data for 2001 and the previously cited initial parameter values, the minimum of chi-square is reached after 30 Levenberg-Marquardt iterations for both genders and 22 for males.

It is important to note that the program Origin calculates the chi-square values in a non-conventional way, given by the Equation 7:
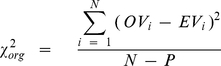
(7)where 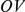 is the observed value, 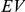 is the expected value, 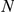 is the number of data points and 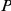 is the number of parameters of the model allowed to be free during the fitting section. As this form is not suitable for p-value estimations, we calculated the conventional chi-squares using the Origin's tools “Set column values” and “Statistics on columns” using the following expression:
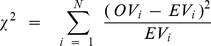
(8)


In all fitting sections, the p-values were estimated from the conventional chi-squares (given by Equation 8) and the respective degrees of freedom, which are the number of data points minus the number of free parameters in such fitting sections.

## Supporting Information

Material S1 **The parameter values that were presented in this preliminary article can be used in the program initialization.**
(PDF)
