# Supplementary material for: The Threshold Bias Model: A Mathematical Model for the Nomothetic Approach of Suicide
Source: PLoS One. 2011 Sep 1;6(9):e24414. doi: 10.1371/journal.pone.0024414 (PMC3164734; doi:10.1371/journal.pone.0024414)
Supplement: Material S1 — The parameter values that were presented in this preliminary article can be used in the program initialization. (PDF) [file pone.0024414.s001.pdf]

SCIENTIA PLENA **VOL. 4, NUM. 8** **2008**
www.scientiaplena.org.br

Sobre a evolução da consciência I: A matemática da autodestruição

W. S. D. Folly

Núcleo de Física, Universidade Federal de Sergipe, 49500-000, Itabaiana-SE, Brasil

wfolly@fisica.ufs.br

(Recebido em 01 de junho de 2008; aceito em 14 de agosto de 2008)

Neste trabalho, apresenta-se um modelo matemático para a evolução temporal do nível de consciência humano baseado na Teoria Psicanalítica de Sigmund Freud. Utilizando-se algumas hipóteses iniciais sobre os mecanismos desencadeadores do comportamento suicida, aplicou-se o modelo a dados estatísticos de taxas de suicídios oriundos de diferentes regiões do mundo a fim de obter um perfil hipotético do nível de consciência médio por faixa etária das populações destas regiões.
Palavras-chave: teoria psicanalítica, pulsão de morte, pulsão de vida, nível de consciência, taxas de suicídio.

In this work, is presented a mathematical model to the consciousness time evolution based on the Psychoanalytical Theory proposed by Sigmund Freud. Considering some initial hypotheses about the suicidal behavior, this model was applied to the statistical data of suicide rates observed in different regions of the world. The results allow the evaluation of the hypothetical profiles of consciousness level by age from the respective populations.
Keywords: psychoanalytical theory, death drive, life drive, consciousness level, suicide rates.

---

1. INTRODUÇÃO

Em seu “Projeto para uma Psicologia Científica” de 1895, Sigmund Freud apresentou uma concepção para o funcionamento do aparelho psíquico tomando emprestado parte do formalismo característico da física de sua época. Porém, devido a sua declarada limitação em relação ao formalismo desta ciência [1], Freud acabou por criar uma concepção para o aparelho psíquico sem prestar uma obediência cega ao embasamento formal da Física. Existem, porém, vários conceitos teóricos da Teoria Psicanalítica que podem ser diretamente relacionados a conceitos físicos. Como exemplo deste paralelismo entre concepções teóricas, é possível citar a energia de investimento pulsional e sua relação com o conceito de energia potencial elétrica.

O conceito de pulsão e sua teoria é, na opinião do próprio Freud, a parte mais importante e inconclusa de toda a Teoria Psicanalítica [2]. Quem estuda a teoria das pulsões, principalmente na sua segunda versão, onde foi apresentado o novo dualismo pulsional entre as pulsões de vida e pulsões de morte [3], verifica que, em nenhum outro ponto de sua teoria, Freud esteve tão próximo de utilizar explicitamente conceitos e idéias da Física, tais como, força, pressão e energia. Segundo ele próprio, a teoria das pulsões consiste no caminho natural de interação entre a Teoria Psicanalítica e a Biologia [4].

Neste trabalho apresenta-se um modelo matemático que relaciona a evolução temporal do nível de consciência humano com o conceito de energia pulsional, sendo este último tratado como uma energia potencial da Física, uma vez que, sua modelagem como uma forma cinética de energia seria incompatível com a idéia de relativa constância da atuação pulsional (estímulos endógenos) em comparação aos estímulos exógenos, que são transitórios [5].

Partindo-se da hipótese de que o suicídio é um comportamento humano estritamente associado à predominância ocasional das pulsões de morte como estímulo endógeno, bem como, à ocorrência de um certo nível de consciência que permita a percepção, a elaboração e o conflito dos estímulos exógenos oriundos da realidade externa, utilizou-se este modelo matemático na simulação de dados estatísticos de suicídio coletados em diferentes regiões do mundo. Estas simulações possibilitaram a obtenção dos parâmetros envolvidos no modelo e a posterior comparação dos mesmos.

1.1 ABORDAGEM FÍSICA DE PULSÃO E ENERGIA PULSIONAL

Antes de se envidar qualquer esforço na modelagem matemática do conceito psicanalítico de pulsão, é preciso delimitar exatamente o campo no qual pretende-se atuar, pois, somente uma ação cautelosa pode evitar imposturas intelectuais que freqüentemente decorrem da introdução de uma metodologia utilizada nas ciências matemáticas em uma ciência humana. Entretanto, não pretende-se enveredar por uma discussão profunda do conceito psicanalítico de pulsão, uma vez que, este assunto tem sido vastamente discutido por outros autores. Assim, nesta seção, deter-se-á apenas no que for necessário ao embasamento teórico das novas proposições.

Considerando-se os textos de vários autores sobre a Teoria Psicanalítica e a própria obra de Freud, verifica-se que o conceito de pulsão ora se aproxima do conceito de força física (quando se atribui a ela características típicas de uma grandeza vetorial como direção e sentido de atuação), ora se confunde com sua própria energia de investimento. Neste trabalho serão adotados os devidos cuidados a fim de não se confundir a pulsão (*trieb*) com sua energia (*triebenergie*), adotando-se esta última como a grandeza de maior interesse para fins de correlação com as idéias da Física.

O motivo de se preferir uma modelagem que envolva o conceito de energia pulsional ao invés da própria pulsão é o mesmo que justifica o uso de um formalismo escalar na Física: a maior simplicidade. Na Física, mais especificamente, na mecânica clássica newtoniana, que é vetorial, para um sistema de N partículas e três graus de liberdade, precisa-se resolver 3N equações de movimento enquanto que, nas versões lagrangeana e hamiltoniana, que são formalismos escalares baseados no conceito de energia, temos que lidar com apenas N equações. Este fato, consiste na principal vantagem do uso destes formalismos nos casos de problemas mecânicos de maior complexidade.

Na Teoria Psicanalítica, o conceito de pulsão de morte ou Thanatos, se refere à força ou pressão (*drang*) que impele novamente o organismo (enquanto matéria) ao seu estado inanimado e inorgânico inicial, do qual este foi afastado pela realidade externa [6]. Freud admitia que as pulsões de morte estavam sempre presentes em maior ou menor intensidade, desde os primeiros anos de vida. Já as pulsões de vida ou Eros tem como missão garantir que o caminho em direção à morte se cumpra de forma suave, sem descontinuidades, ou seja, é função de Eros garantir que o organismo morra à sua própria maneira, evitando a todo custo a morte por causas externas. Estas duas correntes pulsionais nunca são encontradas puras, desconexas entre si. Elas atuam sempre fusionadas em maior ou menor proporção [7].

No que diz respeito às energias de investimento, Freud nomeou simplesmente como libido a energia das pulsões de vida, enquanto que, na concepção atribuída a Weiss [8], libido se refere à energia das pulsões de vida enquanto que destrudo, um termo pouco utilizado atualmente na Psicanálise, nomeia a energia atribuída às pulsões de morte. Neste trabalho, será adotada a denominação proposta por Weiss, ou seja, o termo libido será utilizado em referência à energia investida para unir, para construir, tanto o orgânico quanto o psíquico, enquanto que destrudo corresponderá à energia investida para desunir, para desconstruir o orgânico e o psíquico.

Independentemente das denominações adotadas, as energias pulsionais foram consideradas por Freud como sendo energias físicas [9], apesar de não ser possível medi-las (ainda) como usualmente ocorre no mundo dos fenômenos físicos. Como já mencionado anteriormente, tanto a energia associada às pulsões de morte (destrudo), quanto a associada às pulsões de vida (libido), são concebidas na Teoria Psicanalítica como sendo formas de energias potencial. Associando-se esta idéia àquela que diz que a libido é a energia pulsional investida na construção da psique enquanto que destrudo é a energia investida na desconstrução da psique de modo a levar a matéria novamente ao seu estado inanimado inicial, é possível supor que a libido estaria ligada a uma forma de potencial atrativo enquanto que destrudo seria relacionada a um potencial repulsivo entre as partículas (átomos e moléculas) que constituem órgãos e tecidos de nosso organismo, uma vez que a fonte (*Quelle*) de toda pulsão encontra-se no orgânico [10].

Outro ponto de suma importância que se deve considerar, antes de qualquer tentativa de modelagem matemática, reside no dilema: É o sistema em questão definido por entidades matematizáveis? A resposta a esta pergunta no caso da mente deve ser vista com cautela. Para que uma entidade seja matematizável, em primeiro lugar ela deve ser quantificável de alguma

forma e, de preferência, mensurável. No caso das pulsões, ainda que se tenha o entendimento de se tratar de energias em quantidades variáveis, estas não podem ser medidas. Então, como deve-se proceder? Freud, em seu artigo "As neuropsicoses de defesa" de 1894, diz o seguinte, a respeito deste dilema: "...nas funções psíquicas cabe distinguir algo (quota de afeto, soma de excitação) que tem todas as propriedades de uma quantidade – embora não tenhamos meio de medi-la; algo que é capaz de aumento, diminuição, deslocamento e descarga, e que se difunde pelas marcas mnêmicas das representações como faria uma carga elétrica pela superfície dos corpos" [11]. Assim sendo, esta afirmação de Freud será considerada, neste trabalho, como o ponto de partida para a modelagem matemática das entidades que, apesar de não poderem ser mensuradas diretamente, deixam patentes suas naturezas intensivas, ou seja, de algo que se pode imaginar como quantificável.

Quanto à evolução das pulsões ao longo do tempo de vida do sujeito, Freud diz muito pouco em sua obra. No entanto, é possível tecer certas hipóteses a respeito de como as pulsões e suas respectivas energias evoluem com o tempo.

A Teoria Psicanalítica nos diz que a pulsão surge no início da vida como desvio do instinto de autoconservação que impulsiona o bebê a sugar o seio da mãe pela primeira vez com o intuito de satisfazer sua necessidade de alimento. Neste primeiro contato, o bebê, ao ter sua mucosa oral estimulada pelo contato com seio materno, passa a buscar neste seio, além do alimento, a satisfação de um desejo de ordem sexual, o que dá início à chamada fase oral do desenvolvimento.

Freud identifica cinco fases no desenvolvimento psicossexual humano: a oral, a anal-sádica, a fálica, a fase de latência e a genital, sendo que esta última é normalmente atingida no início da puberdade. Na transição entre estas diferentes fases, para que se verifique uma resolução satisfatória das mesmas, é necessário que ocorra a migração do foco do prazer pelas diferentes zonas erógenas que constituem as diferentes fontes das pulsões parciais, culminando com a organização final das pulsões sexuais focalizadas na região genital ao se atingir a puberdade. É possível então imaginar que a energia pulsional despendida na suplantação das fases pré-genitais a fim de fazer migrar o foco do prazer até a região genital só pode ser atribuída às pulsões de vida, uma vez que, usando a noção de "apoio" no instinto de auto-conservação, visam o objetivo biológico da reprodução, que é indispensável à sobrevivência da espécie. Como diz Freud: "A pulsão sexual está agora subordinada à função reprodutora; tornou-se, por assim dizer, altruística" [12]. Com isso, pode-se supor que, no jogo da fusão e desfusão pulsional, existe uma predominância das pulsões de vida sobre as de morte durante a infância, especialmente durante a organização da sexualidade, afinal, a pulsão de vida sempre atua no sentido de organizar, construir e unir.

Uma vez atingida a fase genital e a idade adulta, pode-se supor que, em um indivíduo normal, a pulsão fusionada resultante do antagonismo entre Eros e Thanatos, permanece mais ou menos bem equilibrada, variando em proporções aproximadamente iguais das duas correntes pulsionais, até que, ao se atingir a senilidade avançada, na resultante das pulsões começam a predominar as pulsões de morte, culminando com a inevitável vitória de Thanatos que cumpre enfim sua missão: levar o organismo novamente ao seu estado inorgânico inicial.

As hipóteses de trabalho utilizadas na modelagem matemática das energias associadas às pulsões de vida (libido) e às pulsões de morte (destrudo) são mostradas resumidamente na tabela 1 abaixo.

Tabela 1: Energias pulsionais e suas características inferidas

**Libido**	**Destrudo**
Energia associada a um potencial médio de caráter atrativo	Energia associada a um potencial médio de caráter repulsivo
Encontra-se presente ao longo de toda a vida do indivíduo, sendo predominante no início	Encontra-se presente ao longo de toda a vida do indivíduo, sendo predominante no final

1.2 POTENCIAIS LIGANTES, HOMEOSTASE E PULSÃO

Para que os átomos, íons e moléculas se organizem nas diferentes estruturas que constituem um organismo vivo, é necessário que para isso haja um favorecimento energético, ou seja, é necessário que existam forças eletrostáticas atrativas que os unam, e, apesar da existência destas forças, o organismo está sempre a perder matéria para o meio externo. Isto ocorre porque os potenciais ligantes que unem entre si átomos, íons e moléculas do organismo nem sempre são suficientemente intensos para sobrepujar outros potenciais ligantes oriundos de átomos e moléculas do meio externo. Assim, é possível que a energia potencial ligante de um átomo ou molécula do organismo se torne não ligante, dependendo da proximidade de elementos atratores (outros átomos ou moléculas) externos à estrutura organizada do mesmo.

Uma vez que o organismo está sempre perdendo matéria e energia para o exterior, estas devem ser constantemente repostas, bem como, os danos causados pela realidade externa aos seus tecidos e órgãos devem ser constantemente reparados em uma troca de matéria e energia constante com o meio (seres vivos são sistemas termodinâmicos abertos).

Pode-se identificar na evolução temporal de um ser vivo pluricelular, três diferentes estágios no que diz respeito a sua homeostase e à quantidade de matéria ordenada em seu organismo. No primeiro estágio, o qual tem início na célula ovo e termina no início da vida adulta, a taxa de ordenamento é maior que a taxa de desordenamento, ou seja, o organismo ganha matéria ordenada. No segundo estágio, que ocorre durante a vida adulta do ser, as taxas de ordenamento e desordenamento são aproximadamente iguais, uma se contrapondo à outra. E, no estágio final, que ocorre na senilidade, a taxa de desordenamento supera a taxa de ordenamento causando uma progressiva perda de matéria ordenada no organismo.

No caso específico dos seres humanos, a consideração freudiana de que a fonte (*Quelle*) das pulsões encontra-se nas excitações de origem orgânica leva à suposição de que exista alguma correlação, ainda que muito complexa para que se possa compreender no momento, entre as energias pulsionais, que servem de estímulo endógeno para a atividade psíquica, e a energia potencial ligante média responsável pelo grau de ordem do organismo. Conseqüentemente, é possível imaginar uma ligação entre pulsão de vida e a maior taxa de ordenamento (orgânico e psíquico) durante o início da vida, bem como, da pulsão de morte e a maior taxa de desordenamento no final da vida de um indivíduo.

2. METODOLOGIA

Considerando-se que o nível de consciência de um indivíduo varia continuamente de zero a um valor de ápice e que esta instância esteja a mercê de estímulos endógenos (energias pulsionais fusionadas) e estímulos exógenos que são recebidos do mundo exterior através do próprio sistema percepção-consciência, pode-se supor que a taxa de variação temporal do nível de consciência seja o produto da energia pulsional resultante dos processos de fusão/desfusão entre libido e destrudo e o próprio nível de consciência, uma vez que este último é determinante do quanto se percebe dos estímulos exógenos. Assim, é possível escrever a seguinte equação diferencial para o nível de consciência do indivíduo:

$$V(t,\xi)\ C(t,\xi) = \frac{\partial C(t,\xi)}{\partial t} \qquad (1)$$

onde o operador $V(t,\xi)$ representa as energias pulsionais fusionadas e será chamado a partir de agora de operador freudiano, em alusão ao hamiltoniano da Mecânica Quântica e $C(t,\xi)$ é o nível de consciência, que, como o operador freudiano, é uma função do tempo t e de outras variáveis desconhecidas aqui representadas por ξ.

Considerando-se que $V(t,\xi)$ não dependa explicitamente de $C(t,\xi)$, pode-se resolver a equação diferencial por separação de variáveis:

$$\int \frac{dC(t,\xi)}{C(t,\xi)} = \int V(t,\xi)\,dt \quad \Rightarrow$$

$$C(t,\xi) = C_{ap} \cdot \exp \int V(t,\xi)\,dt \tag{2}$$

onde C_{ap} é o nível de ápice da consciência.

De forma análoga ao operador evolução temporal dependente do tempo da Mecânica Quântica, que é proporcional ao termo:

$$\exp \int \frac{1}{i\hbar} \hat{H}(t)\,dt ,$$

onde $\hat{H}(t)$ é o hamiltoniano do sistema (que precisa ser conhecido), a evolução temporal do nível de consciência dada pela equação 2 só será definida se conhecermos o operador freudiano $V(t,\xi)$.

2.1 O OPERADOR FREUDIANO COMO A SOMA ENTRE LIBIDO E DESTRUDO

A fim de se encontrar uma função matemática que represente a energia das pulsões de morte (destrudo) e pulsões de vida (libido) deve-se considerar as seguintes hipóteses baseadas na Teoria Psicanalítica:

1) Pulsões de vida estão relacionadas com energias ligantes e ordenadoras e pulsões de morte estão relacionadas com energias não ligantes e desordenadoras tendo, portanto, sinais opostos.

2) As duas correntes pulsionais (e suas energias de investimento) estão sempre presentes ao longo da vida fusionadas em maior ou menor proporção.

3) Supostamente existe um predomínio das pulsões de vida sobre as de morte no início da vida.

4) Supostamente existe um predomínio das pulsões de morte sobre as de vida no final da vida.

Assim, é possível imaginar os seguintes gráficos hipotéticos da libido e da destrudo ao longo do tempo de vida de um indivíduo (ver Fig. 1):

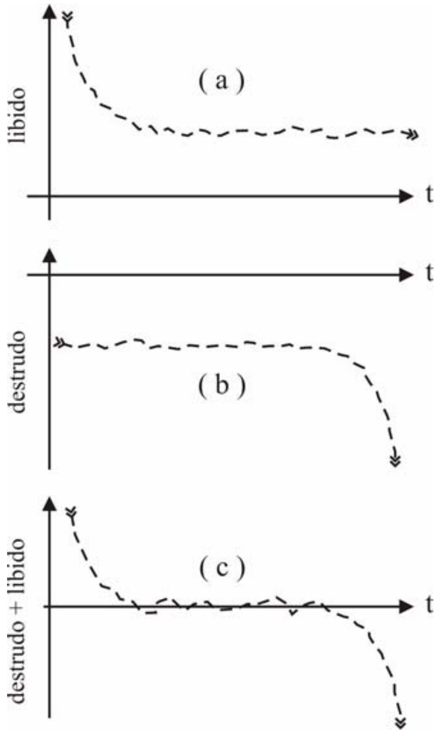

Fig. 1: Gráficos hipotéticos das evoluções temporais da libido (a), da destrudo (b) e do freudiano (soma destrudo + libido) (c) ao longo da vida de um indivíduo. Note que a libido é representada por uma função sempre positiva e a destrudo por uma função sempre negativa.

Como pode ser observado na Fig. 1, espera-se que o freudiano (soma destrudo + libido) apresente uma região positiva no início da vida, devido a preponderância de Eros, e uma região negativa no final da vida, devido à preponderância de Thanatos.

Diferentemente dos problemas da Física, nos quais o hamiltoniano do sistema estudado envolve termos de energia conhecidos, no presente caso não se sabe quase nada sobre o freudiano V, ou seja, tudo não passa de hipóteses especulativas. No entanto, a fim de tornar útil a Eq. 2, é necessário representar V por alguma função do tempo que seja simples, que contenha poucos parâmetros a determinar e que apresente as propriedades mostradas na Fig. 1c. Uma escolha razoável consiste em utilizar alguma função monotonicamente decrescente do tempo como, por exemplo, a função:

$$V = -a \cdot \left(\frac{\beta + 1}{\tau_{1/2}} \right) \cdot \left(\frac{t - \tau_{1/2}}{\tau_{1/2}} \right)^{\beta} \quad (3)$$

onde a, β e $\tau_{1/2}$ são parâmetros a serem determinados empiricamente. Na realidade, os valores destes parâmetros não são de livre escolha. Sabe-se que estes parâmetros devem obedecer a algumas restrições em virtude das propriedades mostradas na Fig. 1c e justificadas nas Figs 1a e 1b. Assim, $a > 0$, β deve ser um inteiro ímpar e $\tau_{1/2}$ deve ser um valor compatível com a metade do tempo médio de vida humano.

2.2 O SUICÍDIO: UM COMPORTAMENTO HUMANO CONSCIENTE ATRIBUÍDO À AÇÃO DE THANATOS

Dentre todos os comportamentos humanos, o suicídio se destaca por estar, em última instância, intimamente relacionado com a consciência e com o caráter da energia pulsional.

É bem verdade que este ato extremo de autodestruição em geral decorre de uma imensa complexidade de problemas sociais, emocionais e ideológicos. Mas também é verdade que, se o suicídio for estudado sob o ponto da vista da Psicanálise, torna-se inegável a ação consciente e terminal provocada pelas pulsões de morte. Sobre esta relação entre suicídio, energia pulsional destrutiva e ação consciente, é o próprio Freud que diz: "...É provável que ninguém encontre a energia mental necessária para matar-se, a menos que, em primeiro lugar, agindo assim, esteja ao mesmo tempo matando um objeto com quem se identificou e, em segundo lugar, voltando contra si próprio um desejo de morte antes dirigido contra outrem." [13].

Outra correlação entre comportamento suicida e consciência é encontrada na própria definição de suicídio de Durkheim: "...todos os casos de morte resultantes direta ou indiretamente de um ato positivo ou negativo da própria vítima sobre si mesma, que sabia que produziria este resultado" [14]. Ou seja, sem um nível mínimo de consciência, não há percepção da realidade externa e, portanto, não há motivação para o ato. Além disso, não há também a possibilidade de ação destrutiva contra si mesmo.

Além da interpretação psicanalítica de que a psique de um suicida se encontra dominada pelas pulsões de morte, um outro fato importante é que, na maioria dos casos, se um indivíduo suicida é dissuadido de suas intenções pouco antes da consumação do ato, ele tende a desistir, a "perder a coragem" de se matar. Isto sugere que a pulsão de morte, que deflagra o ato do suicídio, ocorre como um surto negativo no freudiano V cuja duração tende a ser extremamente curta. Ou seja, nos instantes iminentes ao ato do suicídio, é plausível supor a coincidência entre uma mudança no valor do freudiano V, que se torna repentinamente negativo (ou mais negativo), e um nível elevado da consciência, que pouco se altera momentaneamente. Esta combinação fatal é o que leva então o indivíduo à realização de uma rápida ação consciente de auto-aniquilação de sua percepção da realidade externa (percebida como profundamente desprazerosa).

Assim sendo, é possível extrair informações importantes para nosso modelo matemático dos dados estatísticos relativos às taxas de suicídio por faixa etária, desde que, algumas novas hipóteses sejam incluídas. A saber:

1) Quanto maior for a intensidade das pulsões de morte em relação às de vida, maior a probabilidade de ocorrer um suicídio.

2) Quanto maior o nível de consciência de um indivíduo, maior a probabilidade de ocorrer um suicídio.

3) As duas hipóteses anteriores são necessárias e suficientes para que ocorra um suicídio.

Matematicamente, pode-se então afirmar que a taxa de suicídios é proporcional ao produto entre a intensidade das pulsões de morte no momento do ato e o nível de consciência do indivíduo, ou seja:

$$[\ Taxa\ de\ suicídios\ ] \propto [\ Intensidade\ das\ pulsões\ \ de\ \ morte\ ]\cdot[\ Nível\ \ de\ \ consciência\ ]$$

Escrevendo-se em termos das variáveis que foram anteriormente propostas, é possível supor a taxa de suicídios seja dada pela seguinte expressão:

$$Taxa\ de\ suicídios\ = \begin{cases} 0 & se\ \ (V-b) > 0 \\ -(V-b)\cdot C & se\ \ (V-b) < 0 \end{cases} \tag{4}$$

onde *V* é o freudiano, *C* é o nível de consciência e *b* é a amplitude média dos surtos de pulsão de morte como mostrado na Fig. 2.

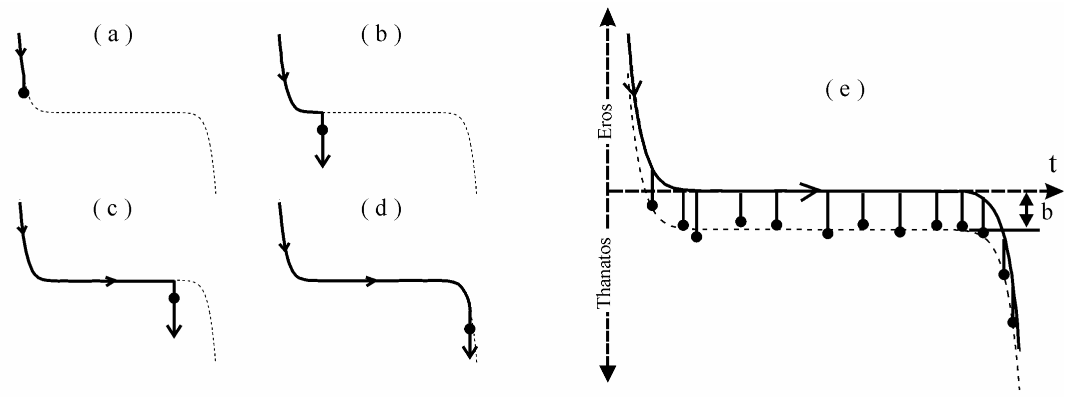

Fig. 2: Em (a), nota-se que a amplitude do surto de pulsão de morte (demarcado com o ponto preto) não leva o operador freudiano a valores negativos e portanto, não potencializa um comportamento suicida do indivíduo como mostrado nos casos (b), (c) e (d). No gráfico (e), a linha tracejada representa o operador freudiano médio fictício deslocado de uma constante b, que representa a amplitude média dos surtos de pulsão de morte deflagradores de suicídios que supostamente ocorrem em um operador freudiano normal (mostrado em linha cheia).

A substituição do freudiano proposto na Eq. 3 na Eq. 2 permite obter o nível de consciência médio:

$$C \;=\; C_{ap}\cdot\exp\left(\int V\,dt\right) \;=\; C_{ap}\cdot\exp\left[-a\left(\frac{t-\tau_{1/2}}{\tau_{1/2}}\right)^{\beta+1}\right] \qquad (5)$$

e, usando-se este resultado na Eq. 4, obtém-se uma expressão da taxa de suicídios na seguinte forma:

$$Taxa\ de\ suicídios \;=\; \qquad (6)$$

$$= \begin{cases} 0 & se \quad t < t_c \\ \left[a\cdot\left(\frac{\beta+1}{\tau_{1/2}}\right)\cdot\left(\frac{t-\tau_{1/2}}{\tau_{1/2}}\right)^{\beta} + b\right]\cdot C_{ap}\cdot\exp\left[-a\left(\frac{t-\tau_{1/2}}{\tau_{1/2}}\right)^{\beta+1}\right] & se \quad t > t_c \end{cases}$$

onde t_c é a solução da equação $-(V-b)\cdot C \;=\; 0$ e representa a idade crítica abaixo da qual a taxa de suicídios é praticamente nula. A existência desta idade crítica é justificada na Fig. 2.

3. RESULTADOS E DISCUSSÃO

3.1 APLICAÇÃO DO MODELO PROPOSTO

Dados estatísticos sobre a mortalidade por suicídios em diferentes regiões e países do mundo podem ser facilmente obtidos na internet (ver referência [15]) . A fim de testar as hipóteses e proposições teóricas iniciais, bem como, determinar os parâmetros que aparecem na Eq. 6, utilizou-se esta última para simular dados estatísticos de taxa de suicídios por faixa etária oriundos de diferentes regiões do mundo em diferentes períodos de tempo.

Na Figs. 3 e 4 respectivamente, são mostradas as taxas de suicídio por 100.000 habitantes nos Estados Unidos da América (média calculada considerando-se ambos os sexos sobre 11 diferentes faixas etárias de 1979 a 1994) [16] e no Sri-Lanka [17] (média calculada considerando-se ambos os sexos sobre 14 diferentes faixas etárias durante o ano de 1950).

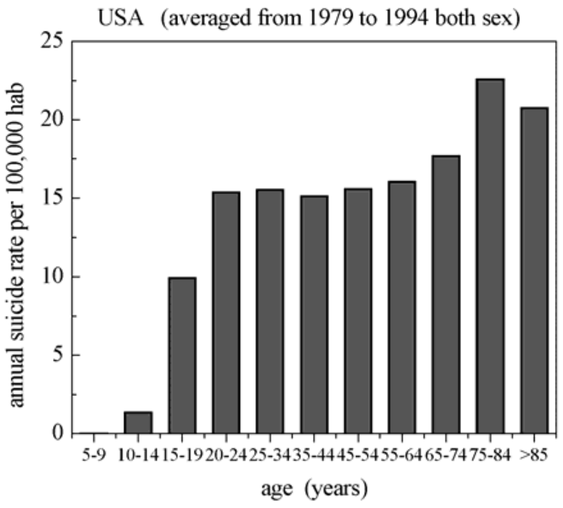

Fig. 3: Taxas anuais de suicídios por faixas etárias nos Estados Unidos da América (Média calculada de 1979 a 1994 para ambos os sexos sobre 11 faixas etárias).

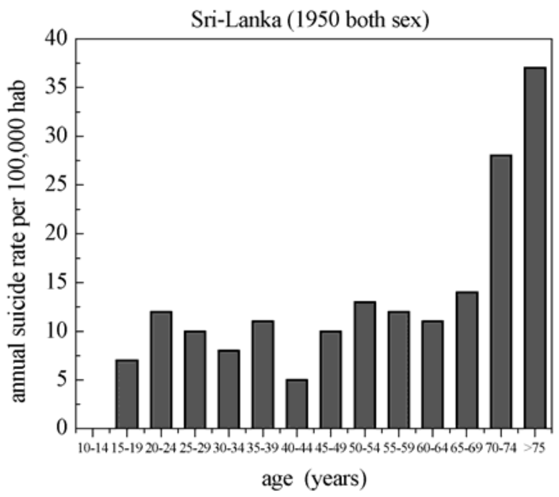

Fig. 4: Taxa anual de suicídios por cem mil habitantes no Sri-Lanka (Média calculada em 1950 para ambos os sexos sobre 14 faixas etárias).

Com auxílio do programa Origin versão 5.0, utilizou-se a Eq. 6 para simular as curvas que mais se aproximam dos dados estatísticos de modo a determinar os valores dos parâmetros C_{ap}, a, b, $\tau_{1/2}$ e β em cada caso. Dentre estes, os parâmetros que têm maior importância são a, b, $\tau_{1/2}$ e principalmente o expoente de β, visto que são diretamente relacionados com a forma da evolução temporal do nível de consciência *C*, do freudiano *V* e, conseqüentemente, das curvas de simulação das taxas de suicídios por faixa etária. Como ainda não se pode medir diretamente o nível de consciência de um indivíduo, o parâmetro C_{ap} (que é um fator multiplicativo) tem uma importância menor, já que sua variação não afeta a forma da curva de simulação obtida.

A Fig. 5 se refere a dados estatísticos oriundos dos Estados Unidos (média calculada ao longo de 15 anos entre 1979 e 1994), incluindo vítimas de ambos os sexos. A Fig. 5a mostra as taxas médias de suicídio por faixas etárias (mostrada na Fig. 3) e a curva simulada mediante a aplicação da Eq. 6 (em linha contínua). As Figs. 5b e 5c mostram, respectivamente, o freudiano médio *V*, o nível de consciência médio *C* e os valores de C_{ap}, a, b, $\tau_{1/2}$ e β utilizados nos cálculos. Já a Fig. 6 se refere aos dados oriundos do Sri-Lanka. São mostrados nesta figura os dados que aparecem na Fig. 4, a curva simulada utilizando-se a Eq. 6 e seus respectivos parâmetros.

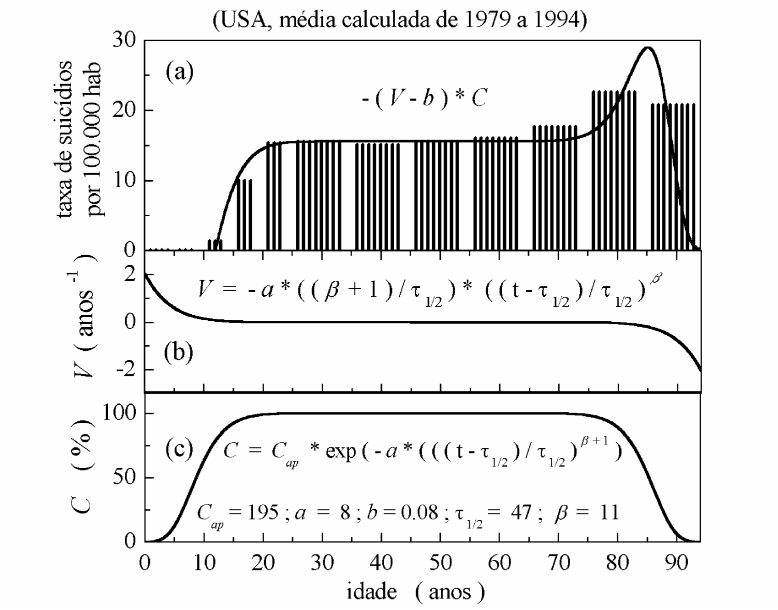

Fig. 5:Taxa de suicídios por faixas etárias nos Estados Unidos da América.Média calculada no período entre 1979 a 1994. Em (a) são mostrados os dados estatísticos apresentados na Fig. 3 e a curva simulada com a aplicação da Eq. 6. Em (b) e (c) são mostrados, respectivamente, o freudiano V e o nível de consciência C, bem como, os valores dos parâmetros de simulação.

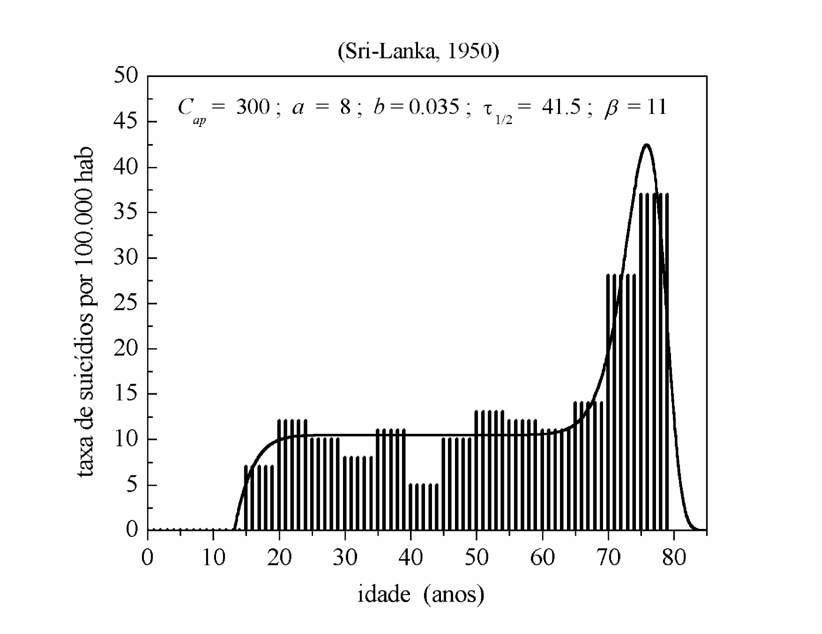

Fig. 6: Esta figura mostra os dados estatísticos da taxa de suicídios por faixa etária no Sri-Lanka relativos ao ano de 1950 (mostrados na Fig. 4), a curva simulada com a aplicação da Eq. 6 e os parâmetros de simulação.

3.2 CASOS ANÔMALOS: A PREVALÊNCIA LIBIDINAL E A PREVALÊNCIA DESTRUDINAL.

Nos dois primeiros conjuntos de dados estudados, admitiu-se que os casos de suicídios foram provocados por surtos de pulsão de morte que, eventualmente, ocorreram em superposição a freudianos bem equilibrados, nos quais os níveis de libido e destrudo são aproximadamente iguais (como mostrados nas Figs. 1 e 2e). Porém, em algumas regiões do mundo e em certas épocas, verifica-se a ocorrência (menos comum) de dados estatísticos que não podem ser aproximados por curvas como as geradas utilizando-se a Eq. 6. Estes dados indicam possíveis desequilíbrios de longa duração nas energias pulsionais, como mostrado na Fig. 7.

Na Fig. 7a é mostrado o gráfico de um freudiano no qual ocorre uma prevalência libidinal constante. Esta prevalência faz com que o nível de consciência atinja seu ápice em idades mais avançadas, como mostrado em 7b e a taxa de suicídios apresenta um único máximo durante a senilidade, como mostrado em 7c. O contrário ocorre no caso de uma prevalência destrudinal como mostrado em 7d. Neste caso, o nível de consciência atinge seu ápice na juventude, como mostrado em 7e e a taxa de suicídios apresenta dois máximos relativos, um durante a juventude e outro em idades avançadas.

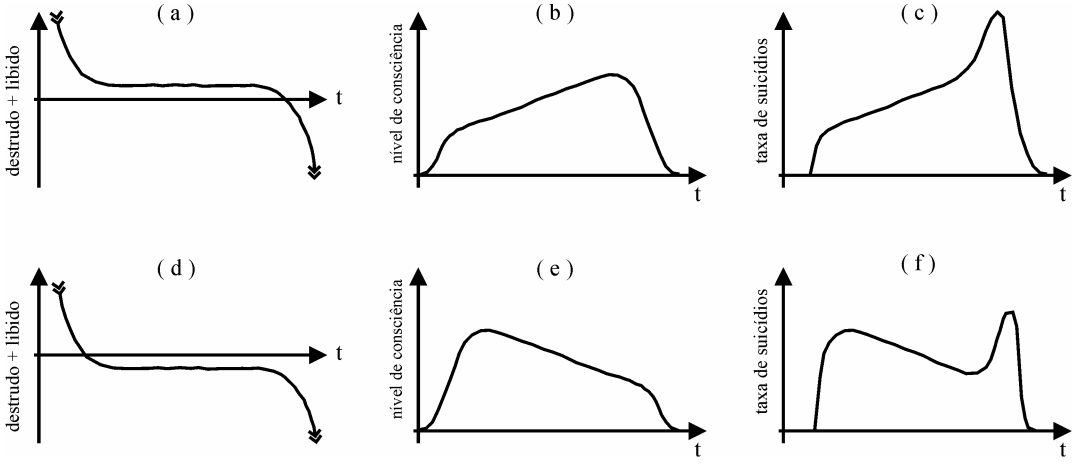

Fig. 7: Possíveis desequilíbrios energéticos na soma destrudo + libido. Em (a) mostra-se a situação de uma sociedade na qual existe uma prevalência libidinal ao longo do tempo. Devido a esta prevalência, o nível de consciência dos indivíduos tende a se elevar com a progressão da idade, como mostrado em (b) e a taxa de suicídios apresenta um único pico na região das idades mais avançadas como mostrado em (c). Já na situação mostrada em (d), existe uma prevalência destrudinal, o que causa uma depleção do nível de consciência dos indivíduos após um certo valor de ápice atingido durante a juventude, como mostrado em (e). Este desequilíbrio faz com que a taxa de suicídios apresente dois máximos, um durante a juventude e outro na senilidade, como se pode notar em (f).

Exemplos de taxas de suicídios por faixas etárias que podem ser explicadas pela prevalência libidinal são as que ocorreram entre homens na França em 1980 [15], como mostrado na Fig. 8, enquanto que as taxas de suicídios no Sri-Lanka em 1999 [17] (mostrada na Fig. 9) podem ser explicadas levando-se em consideração uma prevalência destrudinal.

Nestes casos é necessário introduzir um parâmetro constante V_0 no freudiano (Eq. 3) de forma a dar conta do desvio do mesmo para mais ou para menos. Assim, após a realização da integração da Eq. 5, a taxa de suicídios passa a ser escrita da seguinte forma:

$$\textit{Taxa de suicídios} \;=$$

$$= \begin{cases} 0 & ; \; (t < t_c) \\ \left[a \cdot \left(\dfrac{\beta + 1}{\tau_{1/2}} \right) \cdot \left(\dfrac{t - \tau_{1/2}}{\tau_{1/2}} \right)^{\beta} + b - V_0 \right] \cdot C_{ap} \cdot \exp\left[-a \left(\dfrac{t - \tau_{1/2}}{\tau_{1/2}} \right)^{\beta + 1} + V_0 \, (t - \tau_{1/2}) \right] & ; (t > t_c) \end{cases} \tag{7}$$

Nas Figs. 8 e 9, utilizou-se esta equação para simular as taxas de suicídios ocorridas na França em 1980 e no Sri-Lanka em 1999, podendo-se observar que o valor de V_0 é positivo no caso da França e negativo no caso do Sri-Lanka.

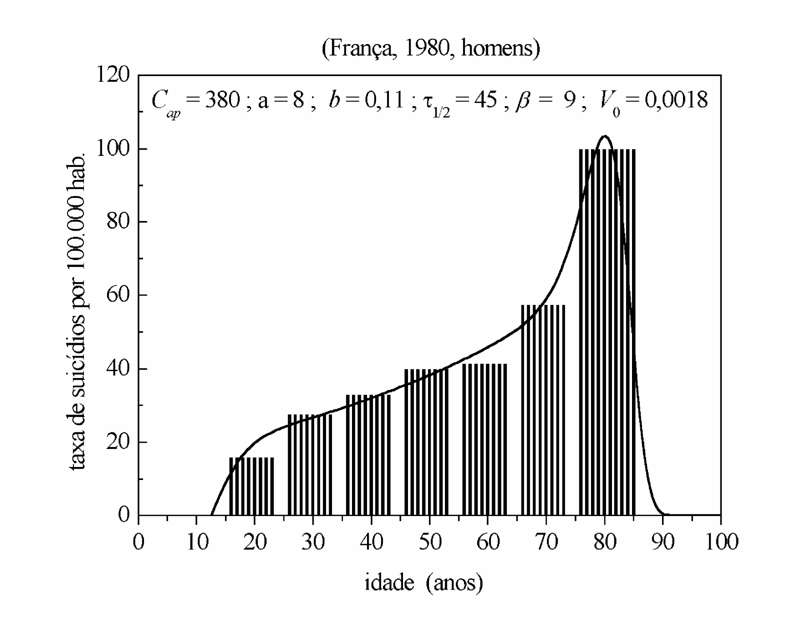

Fig. 8: Taxas de suicídios por faixas etárias na França (homens) em 1980. Exemplo de prevalência libidinal.

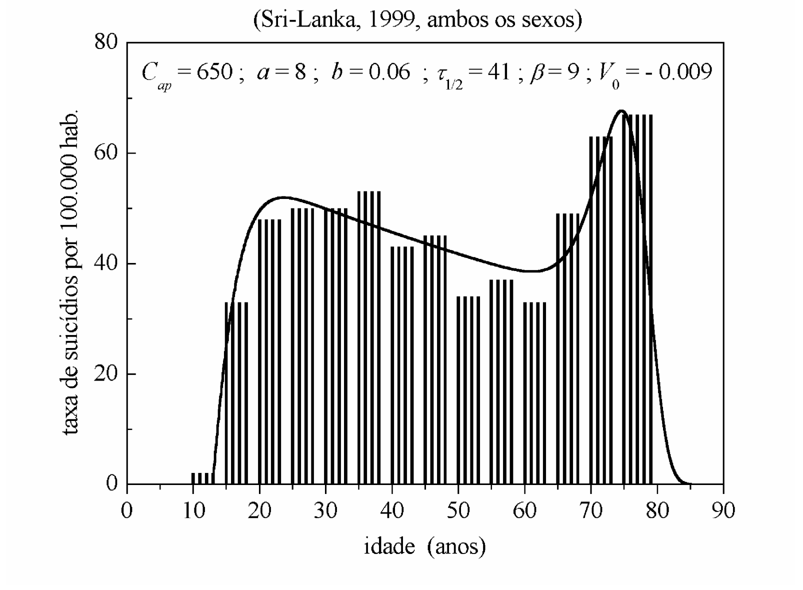

Fig. 9: Taxas de suicídios por faixas etárias no Sri-Lanka em 1999. Exemplo de prevalência destrudinal.

A tabela 2 mostra de uma forma resumida os parâmetros utilizados nas quatro simulações realizadas e os valores da idade crítica t_c, abaixo dos quais as taxas de suicídio são praticamente nulas (obtidos a partir das simulações).

Tabela 2: Parâmetros utilizados nas simulações

**País / período de tempo**	C_{ap} u. a.	a —	b (anos $^{-1}$)	$\tau_{1/2}$ (anos)	β —	V_0 (anos $^{-1}$)	t_c (anos)
EUA / 1979 a 1994	195	8	0,08	47	11	____	12,0
Sri-Lanka / 1950	300	8	0,035	41,5	11	____	13,1
França / 1980	380	8	0,11	45	9	0,0018	12,9
Sri-Lanka / 1999	650	8	0,06	41	9	-0,009	13,0

Pode-se observar na tabela 2 uma grande similaridade entre os valores das idades críticas t_c e dos parâmetros encontrados, principalmente daqueles que influenciam mais fortemente a forma da evolução temporal das taxas de suicídio. Esta similaridade mostra a existência de uma ordem nos dados estatísticos que não pode ser atribuída unicamente a fenômenos de origem social, visto que, os dados estatísticos utilizados neste estudo são oriundos de regiões com enormes diferenças culturais e sociais. Assim, o comportamento suicida, apesar de ser desencadeado pela percepção consciente de fatores que podem ser de ordem social, é um fenômeno que se origina nos recônditos inconscientes da psique individual humana em última instância.

Devido a esta interdependência entre pulsão de morte e nível de consciência, pode-se constatar um fato que os próprios dados estatísticos já evidenciavam, porém, de forma velada: Quanto mais lúcido um indivíduo idoso, mais a mercê dos sofrimentos oriundos dos estímulos exógenos (realidade externa), bem como, dos estímulos destrutivos endógenos (pulsões de morte) o mesmo se encontra. Este fato ressalta a importância das ações individuais, familiares e sociais que visam a minimização do sofrimento e a melhora da qualidade de vida dos indivíduos da terceira idade a fim de minorar as alarmantes taxas de suicídios observadas nestas faixas etárias.

Mostrou-se também que, partindo-se da hipótese da existência de um desequilíbrio energético constante a favor de Eros ou Thanatos no processo de fusão pulsional, é possível explicar os dados estatísticos mais incomuns das taxas de suicídios por faixas etárias, como aqueles mostrados nas Figs. 8 e 9.

Apesar do modelo proposto neste artigo permitir a geração de um perfil hipotético para a evolução temporal do nível de consciência humano ao longo de diferentes faixas etárias, é necessário alertar o leitor para o fato de que, quaisquer tentativas de interpretação destes resultados como sendo válidos em casos individuais, podem incorrer em casos de "falácia ecológica de Robinson" [18]. Isto decorre do fato de que a utilização de dados estatísticos extraídos de grandes populações, durante longos períodos de tempo, concomitantemente com a utilização de hipóteses extremamente simplificadoras da realidade individual, encobrem muitas das idiossincrasias da psique do indivíduo.

Através de estudos mais detalhados das taxas de suicídio por faixas etárias (utilizando-se maiores números de intervalos etários mais estreitos, por exemplo) provavelmente seria possível determinar de forma mais acurada os valores dos parâmetros utilizados na modelagem semi-empírica aqui apresentada, bem como, verificar de forma mais satisfatória suas condições de validade.

Infelizmente, os dados de taxas de suicídio por faixas etárias no Brasil são muito escassos, o que diminui sua importância estatística e dificulta a aplicação do modelo apresentado neste

artigo. Assim, para futuras investigações, pode-se também propor um estudo mais intensivo do suicídio no Brasil utilizando-se esta modelagem semi-empírica a fim de levantar novos questionamentos teóricos sobre o tema.

Agradecimentos

A todos os meus amigos e amigas que contribuíram indiretamente com este trabalho através de indicações bibliográficas, bem como, de frutíferas discussões sobre a Teoria Psicanalítica.

---

1. GARCIA-ROZA, Luiz Alfredo. *Freud e o Inconsciente.* 21a ed. Rio de Janeiro: Jorge Zahar Editor, 2005, p. 46.
2. GARCIA-ROZA, Luiz Alfredo. *Introdução à Metapsicologia Freudiana.* Vol. 3. 6a ed. Rio de Janeiro: Jorge Zahar Editor, 2004, p. 81.
3. FREUD, Sigmund. Além do Princípio do Prazer. In: *Edição Standard Brasileira das Obras Completas de Sigmund Freud. Vol. XVIII.* Rio de Janeiro: Ed. Imago, 1976.
4. GARCIA-ROZA, Luiz Alfredo. *Introdução à Metapsicologia Freudiana.* Vol. 3. 6a ed. Rio de Janeiro: Jorge Zahar Editor, 2004, p. 254-255.
5. GARCIA-ROZA, Luiz Alfredo. *Introdução à Metapsicologia Freudiana.* Vol. 3. 6a ed. Rio de Janeiro: Jorge Zahar Editor, 2004, p. 88.
6. GARCIA-ROZA, Luiz Alfredo. *Freud e o Inconsciente*. 21a ed. Rio de Janeiro: Jorge Zahar Editor, 2005, p. 136-137.
7. GARCIA-ROZA, Luiz Alfredo. *Freud e o Inconsciente*. 21a ed. Rio de Janeiro: Jorge Zahar Editor, 2005, p. 138.
8. WEISS, Edoardo. A Comparative Study of Psycho-analytical Ego Concepts. In: *International Journal of Psycho-Analysis*, v. 38, p. 209-222, 1957.
9. GARCIA-ROZA, Luiz Alfredo. *Introdução à Metapsicologia Freudiana.* Vol. 3. 6a ed. Rio de Janeiro: Jorge Zahar Editor, 2004, p. 260.
10. FREUD, Sigmund. *Edição Standard Brasileira das Obras Completas de Sigmund Freud.* Vol. XIV. Rio de Janeiro: Ed. Imago, 1974, p. 143.
11. GARCIA-ROZA, Luiz Alfredo. *Introdução à Metapsicologia Freudiana.* Vol. 3. 6a ed. Rio de Janeiro: Jorge Zahar Editor, 2004, p. 236.
12. FREUD, Sigmund. Três Ensaios Sobre a Sexualidade. In: *Edição Standard Brasileira das Obras Completas de Sigmund Freud.* Vol. VII. Rio de Janeiro: Ed. Imago, 1972, p. 213.
13. FREUD, Sigmund. A Psicogênese de um Caso de Homossexualismo Numa Mulher. In: *Edição Standard Brasileira das Obras Completas de Sigmund Freud.* Vol. XVIII. Rio de Janeiro: Ed. Imago, 1969, p. 202.
14. THOMPSON, K. *Emile Durkheim*. London: Tavistock Publications, 1982, p. 110.
15. Dados estatísticos da mortalidade por suicídio em diversos países do mundo. Disponíveis em: http://www.suicidemethods.net/tables/int70-88.htm
16. Dados estatísticos da mortalidade por suicídio nos Estados Unidos da América. Disponíveis em: http://www.suicidemethods.net/tables/allageyr.htm
17. Dados estatísticos da mortalidade por suicídios no Sri-Lanka. Disponíveis em: http://www.statistics.gov.lk/social/social%20conditions.pdf
18. ROBINSON, W. S. Ecological Correlations and the Behavior of Individuals. In: *American Sociological Review*, v. 15, p. 351-357, 1950.
